# Supplementary material for: In vivo regulation of an endogenously tagged protein by a light-regulated kinase
Source: G3 (Bethesda). 2025 Apr 7;15(6):jkaf073. doi: 10.1093/g3journal/jkaf073 (PMC12135000; doi:10.1093/g3journal/jkaf073)
Supplement: jkaf073_Supplementary_Data [file jkaf073_supplementary_data.zip › Supplemental_Movie_Legends_G3-2025-405815.docx]

**Supplemental Movie Legends**

**Movie S1.** The control embryo shows normal dorsal closure.

**Movie S2.** Expression of OptoRok in embryo under light exposure leads to abnormal dorsal closure. White arrows indicate points of distortions.

**Movie S3.** Expression of OptoRok in embryo in the dark shows normal dorsal closure.

**Movie S4.** Expression of OptoRok in embryo kept in the dark followed by light activation during imaging results in abnormal dorsal closure. White arrows indicate points of distortions.

The colour scheme and the genotype of the embryos shown in the movies are described in Fig.3 legend.
